# Association Between Intermittent Hypoxia and Left Ventricular Remodeling in Patients With Obstructive Sleep Apnea-Hypopnea Syndrome

**DOI:** 10.3389/fphys.2020.608347

**Published:** 2021-02-12

**Authors:** Ming Deng, Yi-teng Huang, Jian-qing Xu, Xiao Ke, Yi-fei Dong, Xiao-shu Cheng

**Affiliations:** ^1^Department of Cardiology, Fuwai Hospital, Chinese Academy of Medical Sciences, Shenzhen, China; ^2^Department of Cardiovascular Medicine, The Second Affiliated Hospital of Nanchang University, Nanchang, China; ^3^Huazhong University of Science and Technology Union Shenzhen Hospital, Shenzhen, China

**Keywords:** obstructive sleep apnea-hypopnea syndrome, left ventricular remodeling, intermittent hypoxia, oxygen desaturation index, apnea-hypoxia index

## Abstract

The present study was undertaken to examine the association between intermittent hypoxia and left ventricular (LV) remodeling and explore which parameter of intermittent hypoxia is most relevant to LV remodeling in patients with obstructive sleep apnea-hypopnea syndrome (OSAHS). Two hundred eighty six patients underwent polysomnographic examination were enrolled. Based on apnea-hypoxia index (AHI), patients were divided into no, mild, moderate and severe OSAHS groups. Between-group differences in LV remodeling and the association between parameters of intermittent hypoxia and LV remodeling was evaluated. Patients with severe OSAHS were more likely to have hypertension, and higher values of LV mass (LVM) and LVM index (LVMI). In univariate regression analysis, male, body mass index (BMI), systolic and diastolic blood pressure (BP), statins, antihypertensive drugs, creatinine, and parameters of intermittent hypoxia (AHI, obstructive apnea index [OAI], lowest oxygen saturation [LSpO_2_], oxygen desaturation index [ODI], time spent below oxygen saturation of 90% [TS90%], and mean nocturnal oxygen saturation [MSpO_2_]) were associated with LVMI. After multivariate regression analyses, only male gender, BMI, systolic BP, creatinine, and ODI remained significantly associated with LVMI. Compared to those without LV hypertrophy (LVH), patients with LVH had higher ODI. Compared to patients with normal LV, concentric remodeling and eccentric LVH, those with concentric LVH had higher ODI. In conclusion, intermittent hypoxia was significantly associated with left ventricular remodeling; and among various parameters of intermittent hypoxia, ODI was the most relevant to LV remodeling.

## Introduction

With the epidemic of overweight and obesity (Twig et al., 2016; Mouton et al., 2020), the incidence and prevalence of obstructive sleep apnea-hypopnea syndrome (OSAHS) is continuously increasing around the world including China (Cai et al., 2017; Osman et al., 2018). Numerous studies have demonstrated that OSAHS patients have a higher risk of target organ damage (TOD), cardiovascular disease, and all-cause mortality even after adjustment for traditional risk factors and comorbidities (Wang et al., 2016; Floras, 2018; Perger et al., 2019). Unfortunately, up till now, despite promising results in observational studies, there is no evidence from randomized controlled trials demonstrating that OSAHS management by continuous positive airway pressure (CPAP) can improve clinical outcomes (McEvoy et al., 2016; Da Silva Paulitsch and Zhang, 2019; Patil et al., 2019). Therefore, better understanding the mechanisms by which OSAHS causes TOD may help to prevent disease progression and reduce cardiovascular events and mortality.

Left ventricular hypertrophy (LVH) is a sensitive and specific marker of left ventricular remodeling (Huang et al., 2017; Sha et al., 2017). LVH progression results in the development of congestive heart failure, which is associated with great health and economic burdens (Yancy et al., 2013; Ponikowski et al., 2016; Cai et al., 2018). Prior studies have shown that the prevalence of LVH is higher in people with OSAHS than their counterparts without OSAHS (Sekizuka et al., 2017; Hanlon et al., 2019; Maitikuerban et al., 2019). In addition, there is a linear relationship between the severity of OSAHS and LVH (Cuspidi et al., 2019, 2020; Krasińska et al., 2019). The mechanisms are partly attributable to OSAHS-induced blood pressure elevation and intrathoracic mechanical pressure increase (Cai et al., 2016; Cuspidi et al., 2019, 2020; Krasińska et al., 2019). Notably, due to intermittent obstruction of upper respiratory airway, people with OSAHS experience intermittent hypoxia, which causes reactive oxygen species production and endothelial dysfunction (Perrini et al., 2017; Sozer et al., 2018). Numerous studies have reported the adverse effects of intermittent hypoxia on cardiovascular system (Beaudin et al., 2017; Perrini et al., 2017; Bostanci et al., 2018), however, whether there is an independent association between intermittent hypoxia and LVH is unknown. In addition, which parameter of intermittent hypoxia is the most clinically relevant to LVH development is also unknown. This entity is important because if confirmed, intervention to intermittent hypoxia may help to retard LV remodeling progression and prevent LVH development, which in turn reduces the incidence of congestive heart failure. Furthermore, the most relevant parameter of intermittent hypoxia identified can be used as a specific marker to quantify the risk of developing LVH.

Herein, leveraging data from our ongoing prospective cohort study, we conducted a cross-sectional analysis to evaluate that: (1) the association between intermittent hypoxia and LV remodeling; (2) which parameter of intermittent hypoxia is most relevant to LV remodeling in patients with OSAHS.

## Materials and Methods

### Study Participants

**Figure 1:**
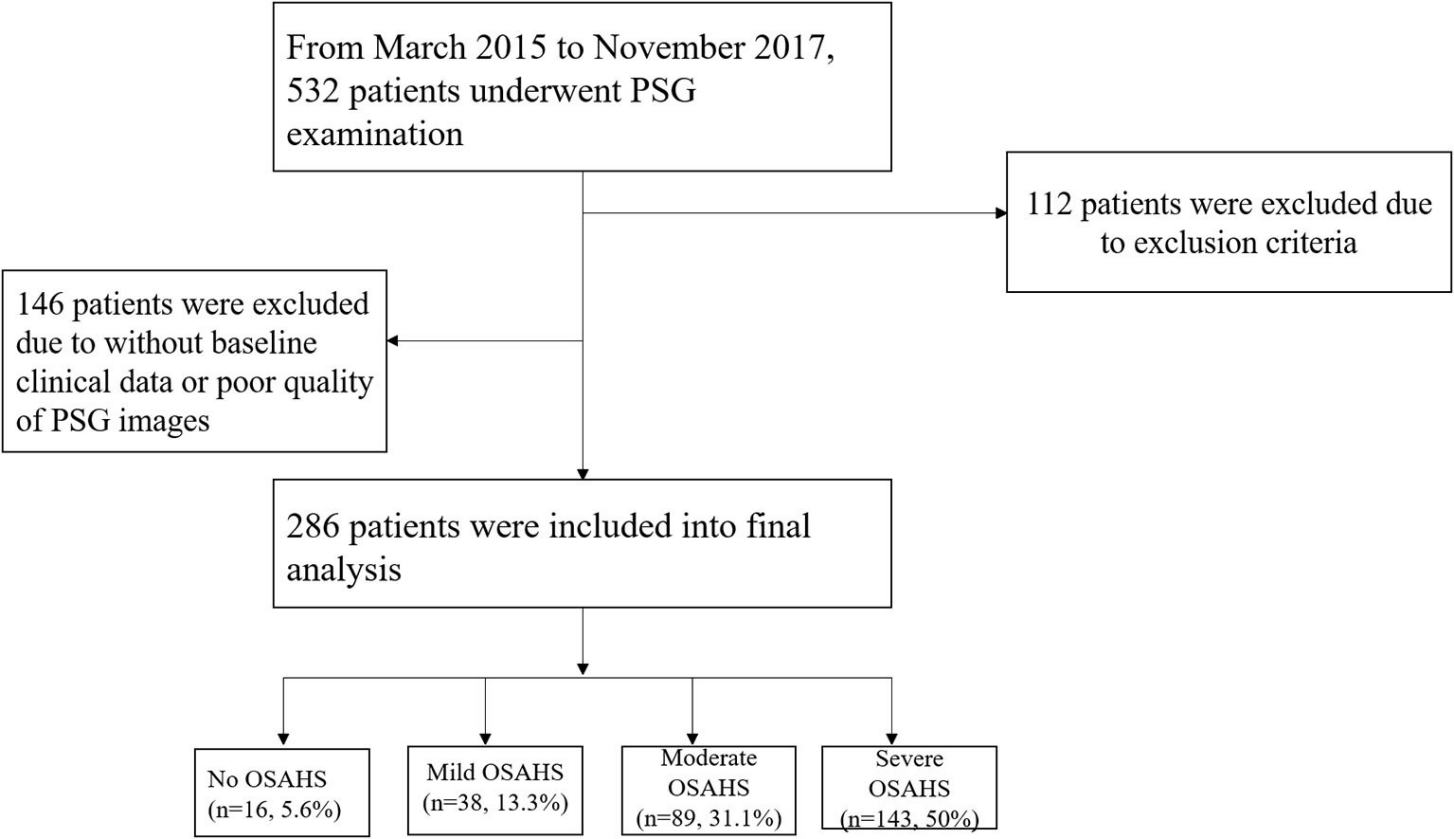
Study flowchart. OSAHS, obstructive sleep apnea-hypopnea syndrome; PSG, polysomnography.

Participants who were admitted to our hospital for polysomnography (PSG) examination were screened, and the exclusion criteria were as follow: (1) prior diagnosis of central sleep apnea; (2) secondary hypertension except for OSAHS-induced hypertension; (3) coexisting cardiovascular disease which could influence left ventricle structure and/or function: coronary heart disease, rheumatic heart disease, congenital heart disease, cardiomyopathy, or left ventricular ejection fraction <50%; (4) coexisting pulmonary disease: chronic obstructive pulmonary disease, acute asthma, moderate to severe pleural effusion, interstitial lung disease, or severe pulmonary infection; (5) the duration of PSG examination <7 h or poor quality of PSG images; (6) lack of important baseline clinical data. Between September 2015 and November 2017, a total of 286 people who had underwent PSG examination and qualified for the study criteria were enrolled (Figure 1). The scoring for obstructive apneas and hypopneas attributed to either central or obstructive events was performed according to The AASM Manual for the Scoring of Sleep and Associated Events Version 2.4 (Berry et al., 2017). The current study was approved by the Clinical Research Ethic Committee of the Second Affiliated Hospital of NanChang University. Signed informed consent was obtained before participant's enrollment.

### Baseline Characteristics Collection

Baseline characteristics, including age, sex, body weight and height, smoking status and alcohol consumption, prior medical history and current medications used, were collected via the electronic health record. Fasting venous blood was drawn for the analysis of uric acid, creatinine, blood urea nitrogen (BUN), lipid panel, fasting plasma glucose (FPG) and homocysteine. Body mass index (BMI) was calculated using weight in kilograms divided by height in squared meters, and BMI ≥28 kg/m^2^ was defined as obesity based on the World Health Organization criteria for Asian population (WHO Expert Consultation, 2004). Estimated glomerular filtration rate (eGFR) was calculated using the Modification of Diet in Renal Disease (MDRD) formula.

### PSG Examination

PSG examination was performed to evaluate the apnea-hypopnea index (AHI) using Philips Respironics Alice PDx. Participants were informed about the detailed procedures of PSG examination. Twenty-four hours before PSG examination, sedative medications, alcohol, tea and coffee were not allowed. The total duration of PSG examination must be >7 h. If the airflow was completely blocked for more than 10 s or >50% reduction in respiratory airflow accompanying >3% reduction in oxygen saturation (SaO_2_) for more than 10 s, the apnea or hypopnea events would be recorded. Based on the the AASM Manual for the Scoring of Sleep and Associated Events Version 2.4 (Berry et al., 2017), participants with AHI of 5–15 events/h were defined as mild, 16–30 events/h were moderate, and 30 events/h were severe OSAHS. In addition, other relevant intermittent hypoxia parameters, including obstructive apnea index (OAI), lowest oxygen saturation (LSpO_2_), oxygen desaturation index (ODI), time spent below oxygen saturation of 90% (TS90%), and mean nocturnal oxygen saturation (MSpO_2_) were also collected concurrently.

### Echocardiogram and LVH Evaluation

Echocardiogram was performed by experienced cardiologists using the Phillip iE33. Left ventricular ejection fraction (LVEF), left ventricular internal diameter in diastole (LVIDd), left ventricular internal diameter in systole (LVIDs), interventricular septal thickness (IVS), and left ventricular posterior wall thickness (LVPW) were measured in accordance to the guideline recommendation (Badano et al., 2018; Heart Failure Group of Chinese Society of Cardiology of Chinese Medical Association, 2018). Relative wall thickness (RWT) was calculated as follow: (IVS + LVPW)/LVIDd. Left ventricular mass (LVM) was calculated as follow: 0.8 × {1.04 × [(LVIDd + LVPW + IVS)^3^ – (LVIDd)^3^]} + 0.6 g. The prevalence of obesity was high in current analysis, therefore, LVM index (LVMI) was adjusted for height^2.7^ and the formula was as follow: LVMI-height^2.7^ = LVM/height^2.7^ g/m^2.7^. LVMI-height^2.7^ > 49.2 g/m^2.7^ in men and LVMI-height^2.7^ > 46.7 g/m^2.7^ in women was defined as LVH. Based on the Ganau criteria (Badano et al., 2018), LV remodeling was classified as follows: normal (without LVH and RWT < 0.42), concentric remodeling (without LVH and RWT ≥ 0.42), eccentric hypertrophy (LVH and RWT < 0.42), and concentric hypertrophy (LVH and RWT ≥ 0.42).

### Statistical Analysis

Continuous variables were presented as mean ± standard deviation (SD) or median (interquartile range; IQR), and between-group difference was compared using one-way ANOVA for variables with normal distribution, and variables with skewed distribution were compared using Kruskal-Wallis H. Categorial variables were presented as number and percentage and between-group difference was compared using the Chi-square. Univariate regression analyses and stepwise multivariate regression analyses were performed to evaluate factors associated with LVMI-height^2.7^. All the analyses were performed using the IBM SPSS statistic 22.0 and a *P*-value < 0.05 was considered as statistical significance.

## Results

### Selection of Study Participants

From March 2015 to November 2017, 532 patients underwent PSG examination in the cardiology department, and a total of 286 patients were included into final analysis in accordance to inclusion and exclusion criteria (Figure 1). Among these patients, 5.6, 13.3, 31.1, and 50% were diagnosed as no OSAHS, mild, moderate and severe OSAHS, respectively.

### Baseline Characteristics Comparisons

As presented in Table 1, compared to patients with mild or moderate OSAHS, those with severe OSAHS were older, and had higher BMI and diastolic blood pressure (*P* < 0.05). Patients with severe OSAHS were more likely to have hypertension and to use angiotensin converting enzyme inhibitor/angiotensin receptor blocker (ACEI/ARB; *P* < 0.05). Serum level of uric acid was also higher in patients with severe OSAHS than those with mild or moderate OSAHS (*P* < 0.05). No significant differences in other baseline characteristics were observed.

**Table 1:** Baseline characteristics comparisons.

**Variables**	**Overall (*N* = 286)**	**No OSAHS (*N* = 16)**	**Mild OSAHS (*n* = 38)**	**Moderate OSAHS (*n* = 89)**	**Severe OSAHS (*n* = 143)**
Male, *n* (%)	218 (76.2)	10 (62.5)	27 (71.1)	66 (74.2)	115 (80.4)
Age, years^#^	51 (40, 61)	39 (29, 54)	56 (47, 64)	55 (42, 63)	50 (39, 59)^&^
BMI, kg/m^2^	27.8 ± 4.2	24.5 ± 2.9	26.2 ± 3.3	26.8 ± 3.3	29.2 ± 4.4^&^
SBP, mm Hg^#^	141 (126, 160)	139 (125, 164)	130 (122, 156)	138 (123, 161)	145 (128, 165)
DBP, mm Hg^#^	85 (74, 100)	88 (73, 99)	81 (72, 86)	80 (74, 97)	89 (74, 106)^&^
Smoking, *n* (%)	92 (32.2)	5 (31.3)	9 (23.7)	33 (37.1)	45 (31.5)
Alcohol use, *n* (%)	49 (17.1)	4 (25.0)	5 (13.2)	17 (19.1)	23 (16.1)
HTN, *n* (%)	249 (87.1)	10 (62.5)	28 (73.7)	81 (91.0)^*^	130 (90.9)^*^
DM, *n* (%)	35 (12.2)	2 (12.5)	3 (7.9)	14 (15.7)	16 (11.2)
Af, *n* (%)	17 (5.9)	1 (6.3)	2 (5.3)	4 (4.5)	10 (7.0)
Statins, *n* (%)	142 (49.7)	4 (25.0)	15 (39.5)	46 (51.7)	77 (53.8)
CCB, *n* (%)	185 (64.7)	6 (37.5)	26 (68.4)	60 (67.4)	93 (65.0)
ACEI/ARB, *n* (%)	173 (60.5)	3 (18.8)	21 (55.3)	54 (60.7)	95 (66.4)^&^
Beta-blocker, *n* (%)	90 (31.5)	2 (12.5)	13 (34.2)	27 (30.3)	48 (33.6)
MRA, *n* (%)	23 (8.0)	2 (12.5)	3 (7.9)	9 (10.1)	9 (6.3)
Folate, *n* (%)	55 (19.2)	2 (12.5)	7 (18.4)	16 (18.0)	30 (21.0)
FPG, mmol/L^#^	5.30 (4.74, 6.02)	4.96 (4.22, 6.28)	5.26 (4.51, 5.78)	5.23 (4.73, 5.91)	5.32 (4.81, 6.13)
TC, mmol/L^#^	4.69 (4.07, 5.26)	4.70 (4.34, 5.48)	4.69 (3.90, 5.24)	4.64 (4.00, 5.21)	4.69 (4.09, 5.33)
TG, mmol/L^#^	1.87 (1.29, 2.58)	1.68 (0.81, 2.53)	1.79 (1.34, 2.33)	1.60 (1.20, 2.57)	2.04 (1.42, 2.72)
HDL-C, mmol/L^#^	1.01 (0.88, 1.14)	1.01 (0.95, 1.26)	1.02 (0.91, 1.18)	1.01 (0.88, 1.17)	0.99 (0.87, 1.11)
LDL-C, mmol/L^#^	2.96 (2.47, 3.51)	2.96 (2.59, 3.57)	2.96 (2.30, 3.28)	2.95 (2.44, 3.44)	2.96 (2.48, 3.61)
HCY, umol/L^#^	13.2 (11.2, 15.9)	15.1 (11.7, 16.6)	12.8 (11.0, 15.1)	13.1 (11.1, 16.6)	13.2 (11.2, 15.7)
BUN, mmol/L^#^	5.31 (4.59, 6.50)	5.24 (4.20, 6.30)	5.44 (4.85, 6.45)	5.33 (4.58, 6.73)	5.30 (4.53, 6.40)
Creatinine, umol/L^#^	79.1 (67.3, 91.7)	79.0 (60.0, 88.1)	79.9 (67.6, 90.6)	79.1 (65.6, 92.0)	79.1 (68.2, 91.9)
Uric acid, umol/L^#^	420.3 (342.9, 485.6)	413.6 (293.2, 460.8)	407.4 (348.5, 490.9)	399.8 (324.6, 462.1)	427.1 (362.4, 514.1)^&^
eGFR (mL/min/1.73 m^2^)^#^	111.7 (94.6, 133.6)	118.3 (87.6, 163.9)	108.4 (93.1, 124.7)	111.7 (94.1, 134.5)	112.2 (96.7, 131.6)

*OSAHS, obstructive sleep apnea-hypopnea syndrome; BMI, body mass index; SBP, systolic blood pressure; DBP, diastolic blood pressure; HTN, hypertension; DM, diabetes mellitus; Af, atrial fibrillation; CCB, calcium channel blocker; ACEI/ARB, angiotensin converting enzyme inhibitor/angiotensin receptor blocker; MRA, mineralocorticoid receptor antagonist; FPG, fasting plasma glucose; TC, total cholesterol; TG, triglyceride; LDL-C, low density lipoprotein cholesterol; HDL-C, high density lipoprotein cholesterol; HCY, homocysteine; BUN, blood urine nitrogen; eGFR, estimated glomerular filtration rate*.

& *P < 0.05 vs. mild and moderate OSAHS*.

* *P < 0.05 vs. no OSAHS and mild OSAHS*.

# *Presented as median (interquartile range)*.

### Echocardiographic Parameters Comparisons

As presented in Table 2, compared to patients with mild, moderate and severe OSAHS, right ventricular internal diameter and LVIDd were smaller while the E/A ratio was higher in patients with no OSAHS (*P* < 0.05). Compared to patients without OSAHS, mild or moderate OSAHS, those with severe OSAHS had higher values of IVS, LVIDs, LVM, LVMI-height^2.7^, A wave velocity and e' wave velocity (*P* < 0.05).

**Table 2:** Echocardiographic parameters comparisons.

**Variables**	**Overall (*N* = 286)**	**No OSAHS (*N* = 16)**	**Mild OSAHS (*n* = 38)**	**Moderate OSAHS (*n* = 89)**	**Severe OSAHS (*n* = 143)**
RVID, mm^#^	22.0 (20.0, 24.0)	20.5 (19.3, 22.0)^‡^	22.0 (20.0, 23.0)	22.0 (21.0, 24.0)	22.0 (21.0, 24.0)
IVS, mm^#^	10.0 (10.0, 12.0)	10.0 (9.0, 10.0)	10.0 (9.0, 11.0)	10.0 (10.0, 12.0)	11.0 (10.0, 12.0)^&^
LVPW, mm^#^	10.0 (9.0, 11.0)	9.0 (9.0, 10.0)	10.0 (9.0, 10.0)	10.0 (9.0, 10.0)	10.0 (9.0, 11.0)
LVIDd, mm^#^	48.0 (45.0, 51.0)	44.0 (42.0, 46.8)^‡^	48.0 (44.8, 51.0)	48.0 (45.0, 51.5)	48.0 (45.0, 52.0)
LVIDs, mm	31.3 ± 4.1	28.4 ± 3.1	30.8 ± 4.3	31.3 ± 4.3	31.8 ± 3.9^&^
LVEF, %	63.0 (60.0, 67.0)	65.0 (60.5, 67.0)	62.0 (60.0, 69.3)	63.0 (58.5, 69.0)	63.0 (60.0, 66.0)
LVM, g^#^	171.5 (147.8, 200.3)	139.9 (120, 162.8)	163.8 (143.0, 189.0)	171.5 (149.8, 196.8)	178.2 (153.3, 212.0)^&^
LVMI-height^2.7^, g/m^2.7#^	43.0 (37.4, 50.5)	36.1 (29.2, 39.1)	43.5 (35.4, 45.5)	43.9 (38.4, 48.2)	43.6 (37.6, 53.1)^&^
E, m/s^#^	65.5 (53.0, 80.0)	80.0 (64.8, 81.8)	71.0 (57.0, 88.5)	65.0 (51.5, 78.5)	64.0 (52.0, 78.0)
A, m/s	79.2 ± 17.4	66.3 ± 13.9	79.9 ± 20.1	78.9 ± 16.1	80.7 ± 17.3^&^
e', m/s^#^	6.0 (5.0, 7.2)	7.5 (6.1, 10.7)	6.7 (5.0, 7.6)	6.0 (5.0, 7.0)	5.8 (4.8, 7.0)^&^
a', m/s^#^	9.2 (8.0, 10.5)	9.5 (8.2, 10.3)	9.3 (8.2, 10.5)	9.0 (8.0, 10.8)	9.3 (8, 10.4)
E/e'^#^	11.0 (8.8, 13.3)	9.5 (7.2, 12.2)	11.6 (8.4, 13.6)	10.4 (8.9, 13.3)	11.1 (9.2, 13.3)
E/A^#^	0.8 (0.7, 1.1)	1.2 (0.9, 1.3)^‡^	0.8 (0.7, 1.2)	0.8 (0.7, 1.1)	0.8 (0.7, 0.9)

*OSAHS, obstructive sleep apnea-hypopnea syndrome; RVID, right ventricular internal diameter; IVS, inter-ventricular septum; LVPW, left ventricular posterior wall; LVIDd, left ventricular internal diameter in diastole; LVIDs, left ventricular internal diameter in systole; LVEF, left ventricular ejection fraction; LVM, left ventricular mass; LVMI, left ventricular mass index*.

‡ *P < 0.05 vs. mild, moderate, and severe OSAHS*.

& *P < 0.05 vs. no OSAHS, mild, and moderate OSAHS*.

# *Presented as median (interquartile range)*.

### Polysomnographic Parameters Comparisons

As presented in Table 3, compared to patients without OSAHS, mild or moderate OSAHS, those with severe OSAHS had higher AHI, OAI, ODI, and TS90% (*P* < 0.05), while MSpO_2_ and LSpO_2_ was lower in patients with severe OSAHS (*P* < 0.05).

**Table 3:** Polysomnographic parameters comparisons.

**Variables**	**Overall (*N* = 286)**	**No OSAHS (*N* = 16)**	**Mild OSAHS (*n* = 38)**	**Moderate OSAHS (*n* = 89)**	**Severe OSAHS (*n* = 143)**
AHI, events/h^#^	29.9 (17.4, 49.4)	3.5 (2.5, 4.5)	10.6 (7.4, 12.8)	22.3 (18.1, 26.6)	49.3 (37.3, 61.9)^&^
OAI, events/h^#^	7.5 (2.9, 19.9)	0.4 (0.1, 0.8)	2.3 (1.3, 3.2)	4.7 (2.8, 9.1)	17.6 (8.4, 30.8)^&^
MSpO_2_, %^#^	94 (92, 95)	96 (94.3, 96)	95 (94, 95)	94 (93, 95)	93 (91, 94)^&^
ODI, events/h^#^	22.9 (12.3, 43.7)	3.2 (1.0, 4.0)	6.9 (4.9, 11.4)	16.7 (13.2, 23.2)	43.6 (29.4, 59.2)^&^
LSpO_2_, %^#^	80 (72, 84)	89 (87, 93)	84 (82, 88)	82 (76, 85)	73 (63, 80)^&^
TS90%, %^#^	3.7 (0.9, 15)	0.1 (0, 0.5)	0.6 (0.1, 1.3)	2.4 (0.8, 4.8)	13.7 (3.4, 31.1)^&^

*OSAHS, obstructive sleep apnea-hypopnea syndrome; AHI, apnea-hypopnea index; OAI, obstructive apnea index; MSpO_2_, mean nocturnal oxygen saturation; ODI, oxygen desaturation index; LSpO_2_, lowest oxygen saturation; TS90%, time spent below oxygen saturation of 90%*.

& *P < 0.05 vs. no OSAHS, mild, and moderate OSAHS*.

# *Presented as median (interquartile range)*.

### Factors Associated With LVMI-Height^2.7^

As presented in Table 4, in univariate linear regression analysis, male, BMI, systolic and diastolic blood pressure, hypertension, statins, antihypertensive drugs, BUN, creatinine, eGFR, AHI, OAI, LSpO_2_, ODI, TS90%, and MSpO_2_ were significantly associated with LVMI-height^2.7^. However, after multivariate linear regression analyses, only male, BMI, systolic blood pressure, BUN, creatinine, and ODI remained significantly associated with LVMI-height^2.7^.

**Table 4:** Univariate and multivariate linear regression analyses.

**Variables**	**Univariate**		**Multivariate**		**VIF**
	**β (95% CI)**	***P*-value**	**β (95% CI)**	***P*-value**	
Male	−4.31 (−7.911, −0.708)	0.0192	−7.196 (−10.442, −3.951)	<0.001	1.099
Age	0.046 (−0.072, 0.163)	0.4453	N/A		
BMI	0.9 (0.543, 1.257)	<0.001	0.597 (0.223, 0.97)	0.0019	1.390
SBP	0.161 (0.105, 0.217)	<0.001	0.083 (0.028, 0.137)	0.003	1.157
DBP	0.158 (0.075, 0.24)	<0.001	–		
Smoking	1.18 (−2.132, 4.491)	0.4837	N/A		
Alcohol use	−0.315 (−4.424, 3.793)	0.88	N/A		
HTN	7.117 (2.58, 11.655)	0.0022	–		
DM	−0.788 (−5.511, 3.935)	0.743	N/A		
Af	−0.953 (−7.5, 5.593)	0.7746	N/A		
Statins	3.297 (0.225, 6.37)	0.0355	–		
CCB	5.517 (2.342, 8.691)	<0.001	–		
ACEI/ARB	6.859 (3.795, 9.922)	<0.001	–		
Beta-blocker	7.788 (4.581, 10.996)	<0.001	–		
MRA	13.234 (7.755, 18.713)	<0.001	–		
Folate	5.222 (1.341, 9.103)	0.0085	–		
FPG	0.345 (−0.655, 1.344)	0.4976	N/A		
TC	0.563 (−1.023, 2.15)	0.4852	N/A		
TG	0.274 (−0.83, 1.379)	0.6252	N/A		
HDL-C	3.198 (−3.95, 10.347)	0.3792	N/A		
LDL-C	1.186 (−0.65, 3.023)	0.2046	N/A		
HCY	0.096 (−0.1, 0.292)	0.3373	N/A		
BUN	2.42 (1.627, 3.213)	<0.001	1.332 (0.332, 2.331)	0.0092	1.945
Creatinine	0.097 (0.064, 0.13)	<0.001	0.051 (0.008, 0.095)	0.0198	2.111
Uric acid	0.022 (0.008, 0.036)	0.0017	–		
eGFR	−0.093 (−0.139, −0.048)	<0.001	–		
AHI	0.146 (0.075, 0.218)	<0.001	–		
OAI	0.152 (0.045, 0.259)	0.0056	–		
MSpO_2_	−0.994 (−1.464, −0.525)	<0.001	–		
ODI	0.147 (0.081, 0.212)	<0.001	0.078 (0.009, 0.146)	0.0263	1.410
LSpO_2_	−0.258 (−0.39, −0.127)	<0.001	–		
TS90%	0.157 (0.079, 0.236)	<0.001	–		

*BMI, body mass index; SBP, systolic blood pressure; DBP, diastolic blood pressure; HTN, hypertension; DM, diabetes mellitus; Af, atrial fibrillation; CCB, calcium channel blocker; ACEI/ARB, angiotensin converting enzyme inhibitor/angiotensin receptor blocker; MRA, mineralocorticoid receptor antagonist; FPG, fasting plasma glucose; TC, total cholesterol; TG, triglyceride; LDL-C, low density lipoprotein cholesterol; HDL-C, high density lipoprotein cholesterol; HCY, homocysteine; BUN, blood urine nitrogen; eGFR, estimated glomerular filtration rate; AHI, apnea-hypopnea index; OAI, obstructive apnea index; MSpO_2_, mean nocturnal oxygen saturation; ODI, oxygen desaturation index; LSpO_2_, lowest oxygen saturation; TS90%, time spent below oxygen saturation of 90%; CI, confidence interval; N/A, non-applicable; VIF, variance inflation factor*.

### Comparison of ODI According to LV Remodeling

**Figure 2:**
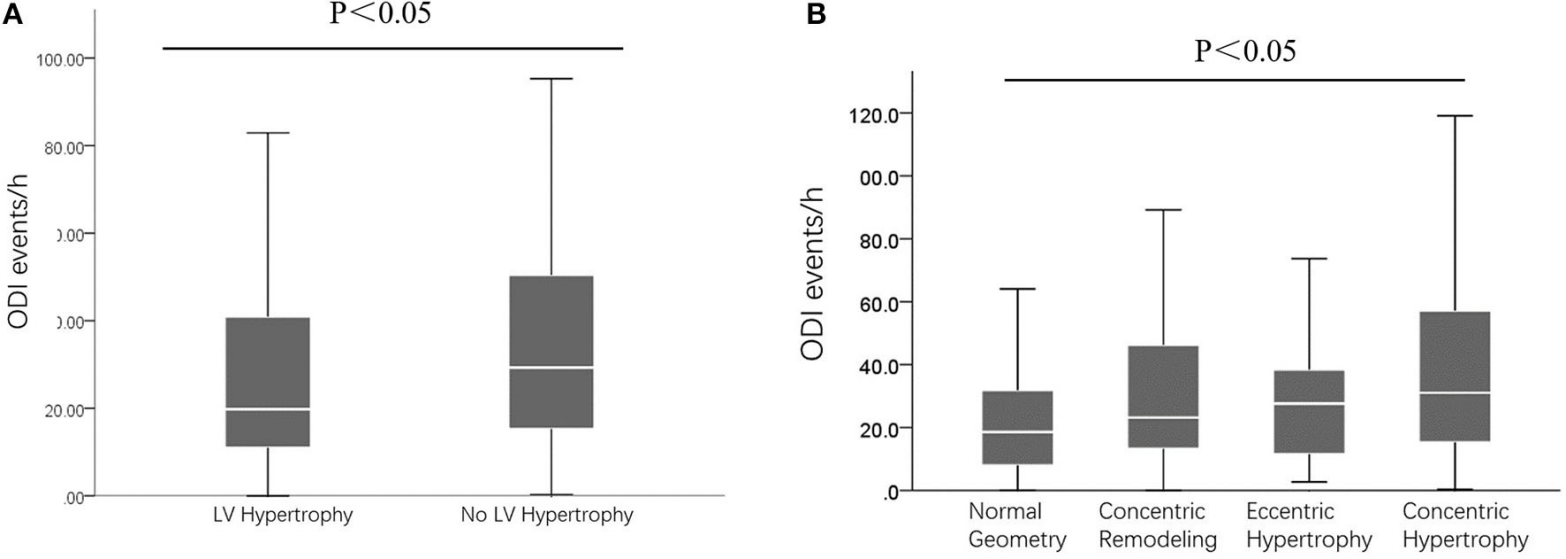
The ODI events between/among different patients. **(A)** Comparisons of ODI between those with and without LV hypertrophy; **(B)** Comparisons of ODI between those with different LV remodeling. ODI, oxygen desaturation index; LV, left ventricular.

As presented in Figure 2A, compared to those without LVH, patients with LVH had higher ODI (*P* < 0.05). In addition, as presented in Figure 2B, compared to patients with normal LV, concentric remodeling, and eccentric LVH, those with concentric LVH had higher ODI (*P* < 0.05).

## Discussion

In the present study, we evaluated the association of intermittent hypoxia and LV remodeling in Chinese populations. There are two main findings of current study. First, intermittent hypoxia was significantly associated with left ventricular remodeling as reflected in increased LVMI-height^2.7^. Second, among various parameters of intermittent hypoxia, ODI was the most relevant to LVMI-height^2.7^. These findings together suggest that intervention of intermittent hypoxia may help to retard LV remodeling progression and prevent LVH development. ODI may be a useful marker to predict LVH development and to reflect the efficacy of intervention for intermittent hypoxia.

OSAHS has been considered as an important comorbidity for a variety of cardiovascular diseases (Wang et al., 2016; Floras, 2018; Perger et al., 2019). Numerous studies have shown that increased AHI, an indicator of OSAHS severity, is independently associated with TOD and cardiovascular diseases. For example, Chami et al. (2008) reported that compared to patients with AHI <5 events/h, those with AHI >30 events/h had 1.8-fold higher prevalence of LVH. Cai et al. (2018) reported that increased AHI was associated with higher prevalence of congestive heart failure event after adjusted for other risk factors. Sun et al. (2014) also reported that compared to patients without OSAHS, those with OSAHS had higher IVS, LVIDs, and LVIDd, reflecting a significant LV remodeling. Consistent to prior reports, the current analysis also showed that the values of IVS and LVIDs were higher in patients with severe OSAHS than those without OSAHS or those with less severe OSAHS. Furthermore, we also observed that the LVM and LVMI-height^2.7^ were also significantly higher in patients with severe OSAHS. These findings together strongly indicate that presence of OSAHS is significantly associated with LV remodeling, which presented in a dose-dependent fashion. Furthermore, the velocity of A wave was faster while the lateral mitral annulus e' wave velocity was lower in patients with severe OSAHS, suggesting an impaired diastolic LV dysfunction, which we considered might be due to LV hypertrophic remodeling. With increasing prevalence and incidence of overweight and obesity, it is prudent to screen people with unrecognized or undiagnosed OSAHS, and timely and effective intervention should be implemented to prevent LV remodeling and associated cardiovascular disease.

Various parameters of intermittent oxygen have been developed to assess the severity of OSAHS. The AHI has been the most common use to diagnose and define the severity of OSAHS in clinical practice. However, there are also some limitations of AHI, and the most important one is that the AHI cannot quantitatively reflect the oxygen desaturation during intermittent hypoxia (Kendzerska and Leung, 2016; Kapoor, 2018; Camporro et al., 2019). In the current study, we included six common use parameters of intermittent hypoxia and evaluated their association with LVMI-height^2.7^. As presented in Table 3, the changes of these six parameters were in accordance to increasing severity of OSAHS. In addition, in the univariate linear regression analysis, all these parameters were significantly associated with LVMI-height^2.7^, suggesting each individual parameter is a relevant marker to LV remodeling. Nonetheless, after adjustment for other potential confounding factors including gender, BMI, blood pressure and among others, only ODI remained significantly associated with LVMI-height^2.7^. These findings highlight that compared to other parameters of intermittent hypoxia, ODI might be the most relevant marker related to LV remodeling. Indeed, results from prior reports also support our findings. For example, Tkacova et al. (2014) reported that in hypertensive patients with OSAHS, only ODI was associated with LV remodeling in multivariate regression model. The reasons for the superiority of ODI in relation to LV remodeling are likely multiple, including as follows (Jung Da et al., 2016; Kim et al., 2016; Myllymaa et al., 2016): first, ODI reflects the frequency of intermittent hypoxia, and increased ODI is directly associated with increased hypoxia-reoxygenation injury, causing endothelial dysfunction, arterial constriction, blood pressure elevation, and LV afterload increase; second, increased ODI is associated with incrementally reduced oxygen supply to cardiac tissue, which leaded to cardiomyocytes necrosis and cardiac fibrosis accumulation; third, increased ODI is also associated with renin-angiotensin-aldosterone axis activation, resulting in LV remodeling and hypertrophy. These several lines of evidence suggest that in patients with OSAHS, assessment and monitoring of ODI might provide valuable information to evaluate LV remodeling progression and LVH development. Up to date, there is no clinical data assessing the effects of direct oxygen supplementation on the clinical outcomes for OSAHS treatment. However, most studies performed the CPAP to indirectly improve the oxygen supplementation, which may improve the LVH in the OSAHS patients. Studies showed that CPAP could reverse the LVH in the patients with severe OSA (Cloward et al., 2003; Shivalkar et al., 2006). Colish et al. (2012) demonstrated that both systolic and diastolic abnormalities in patients with OSA could be reversed as early as 3 months into CPAP therapy, with progressive improvement in cardiovascular remodeling over 1 year. In addition, Marin et al. (2005) showed that CPAP treatment reduced the risk of fatal and non-fatal cardiovascular events in the male patients with OSAHS. The improvement of AHI and LSpO_2_ are the main indicators for assessing treatment outcomes of patients with OSAHS, and our results suggest that the decrease in ODI value may be more effective in predicting the improvement of LVH compared with AHI and LSpO_2_. Further studies are needed to evaluate whether improvement of ODI is associated with improvement of LV remodeling and lower risk of LVH. Beyond the level of oxygen saturation achieved, LVH may be caused by a different background sensitivity of the chemoreflex system to hypoxia, and patients with similar ODI may have different degree of LVH depending on the chemoreflex (Deacon-Diaz and Malhotra, 2018). In addition, hypoxia may drive to chemoreflex mediated adrenergic discharge and thus LVH, which still requires future investigations.

There are some limitations of current study needed to address. First, this is a cross-sectional study, and therefore causal relationship should not be drawn. Second, despite we have extensively adjusted for potential confounding factors, unmeasured and undetected factors might still exist and influence the association of ODI and LVMI-height^2.7^. Third, this is a single-center study conducted in Chinese populations and these findings might not be extrapolated to other racial/ethnic populations. Fourth, all participants did not receive any intervention and therefore these findings might not be applied those received treatment, such as continuous positive airway pressure (CPAP). Fifth, the severity of the sample is large with 50% of patients classified as severe, as the data in this study was mainly collected from the patients suspected with OSAHS and these patients were then subjected to the PSG. Studies from Yamaguchi et al. (2016) showed that the percentage of patients with AHI >30 events/h was ≥63%, which was similar to our study. However, large sample size should be considered in the future studies. Last but not the least, consistent to study design, people with cardiovascular and pulmonary diseases were excluded. Therefore, these findings might not be extrapolated to those with cardiovascular or pulmonary diseases.

## Conclusion

In conclusion, the current study shows that among patients with untreated OSAHS, intermittent hypoxia is significantly associated with LV remodeling; and among various parameters of intermittent hypoxia, ODI was the most relevant to LVH. Future studies are needed to evaluate whether improvement of ODI by CPAP treatment can prevent or ameliorate LV remodeling.

## Data Availability Statement

The raw data supporting the conclusions of this article will be made available by the authors, without undue reservation.

## Ethics Statement

The studies involving human participants were reviewed and approved by the Clinical Research Ethic Committee of the Second Affiliated Hospital of NanChang University. The patients/participants provided their written informed consent to participate in this study.

## Author Contributions

MD and X-sC: conception and design of the research. MD, Y-tH, and J-qX: recruiting the volunteers and performing the clinical experiments. XK and Y-fD: interpreted the results. X-sC: draft the manuscript. All authors read and approved the final manuscript.

## Conflict of Interest

The authors declare that the research was conducted in the absence of any commercial or financial relationships that could be construed as a potential conflict of interest.

## Abbreviations

AHI: apnea-hypopnea index
BMI: body mass index
BUN: blood urea nitrogen
CPAP: continuous positive airway pressure
eGFR: estimated glomerular filtration rate
FPG: fasting plasma glucose
IQR: interquartile range
IVS: interventricular septal thickness
LSpO_2_: lowest oxygen saturation
LV: left ventricular
LVEF: left ventricular ejection fraction
LVH: left ventricular hypertrophy
LVIDd: left ventricular internal diameter in diastole
LVIDs: left ventricular internal diameter in systole
LVM: left ventricular mass
LVPW: left ventricular posterior wall thickness
MDRD: modification of diet in renal disease
MSpO_2_: mean nocturnal oxygen saturation
OAI: obstructive apnea index
ODI: oxygen desaturation index
OSAHS: obstructive sleep apnea-hypopnea syndrome
PSG: polysomnography
RWT: relative wall thickness
SaO_2_: oxygen saturation
SD: standard deviation
TOD: target organ damage
TS90%: oxygen saturation of 90%.
